# Stronger Together: Personality, Intelligence and the Assessment of Career Potential

**DOI:** 10.3390/jintelligence6040049

**Published:** 2018-11-13

**Authors:** Franziska Leutner, Tomas Chamorro-Premuzic

**Affiliations:** Department of Psychology and Language Science, University College London, 26 Bedford Way, London WC1H0AP, UK; t.chamorro@ucl.ac.uk

**Keywords:** intelligence, personality, selection, performance, assessment

## Abstract

Personality and intelligence have a long history in applied psychology, with research dating back more than 100 years. In line, early developments in industrial-organizational psychology were largely founded on the predictive power of personality and intelligence measures vis-à-vis career-related outcomes. However, despite a wealth of evidence in support of their utility, the concepts, theories, and measures of personality and intelligence are still widely underutilized in organizations, even when these express a commitment to making data-driven decisions about employees and leaders. This paper discusses the value of personality and intelligence to understand individual differences in career potential, and how to increase the adoption of theories and tools for evaluating personality and intelligence in real-world organizational contexts. Although personality and intelligence are distinct constructs, the assessment of career potential is incomplete without both.

## 1. Introduction

Within the social sciences, few constructs have demonstrated as much real-world utility as personality and intelligence, particularly when it comes to predicting and explaining individual differences in career-related outcomes [1]. The use of personality and intelligence measures for personnel selection, which provides a quantitative estimate of someone’s career potential, dates back more than 100 years [2,3]. And yet, in an age when it is habitual for organizations across all industries to embrace a data-driven approach to making career-related decisions on their prospective and current employees, the use of scientifically-defensible personality and intelligence tools is far from widespread [4]. Consider that in Human Resources (HR) there are approximately 40 million assessments sold per year—not all valid—yet an estimated 4 billion people work in the world [5]. It is therefore safe to assume that most people go through their careers without ever having their personality or intelligence assessed, at least not through commercial tools. Perhaps more strikingly, even inside large multinational corporations, where HR departments are usually aware of the value of scientific assessment tools, the large majority of HR professionals identify career potential via intuitive and subjective methods, such as a single rating by the employee’s direct line manager [6]. There is also a common tendency within HR to overrate intuitive judgments of people’s career potential [7]. By the same token, the most common approach for vetting the career potential of external candidates is the job interview, which is less predictive, more expensive, and more time-consuming than *state-of-the-art* psychometric assessments.

## 2. Relative Predictive Power of Intelligence

In contrast, the explanatory power of intelligence tests is ubiquitous. In a time of unprecedented concern for a “replication crisis” in psychology, as well as the wider social sciences, the consistent validity of intelligence measures as predictors of career-related outcomes is unparalleled. In what is still the largest and most systematic meta-analytic study of the various predictors of job performance—as well as one of the most widely cited articles in applied psychology—Schmidt and Hunter reported a mean validity of *r* = 0.51 for intelligence. The maximum increase in validity provided by any other method, including interviews, a work sample test, and personality assessments, was 27% (for integrity tests) [8]. Twenty years on there have been hundreds of independent studies, as well as several meta-analyses, that have replicated the predictive power of intelligence tests to forecast future job performance and career success. For example, meta-analytic reviews including over 20,000 studies and 5 million participants reported average validities of *r* = 0.50 between intelligence and job performance [9]. Moreover, intelligence measures can also be used to predict counterproductive work behaviors, which are more common in people with lower intelligence levels [10].

In addition, there are wider societal—not just individual—levels to intelligence. For instance, societies thrive when they are better able to allocate jobs to individuals with the right level of intelligence [11]. And between countries, intelligence is strongly and positively correlated with GDP, economic freedom, and prosperity [12]. Likewise, national differences in scientific, educational, and business achievements can be strongly predicted by average intelligence levels [13].

## 3. Relative Predictive Power of Personality

When employers make inferences about a candidate’s ability to perform and maintain a job, they focus not only on their ability, but also motivation and people-skills [14], which makes personality a key determinant of employability. Indeed, personality is an important indicator of an individual’s personal effectiveness, the way in which they interact with and manage others [15]. Personality is also a consistent predictor of subjective career success, influencing how people evaluate their own careers [16]. For example, people who are stable and conscientious evaluate their own job performance more positively [17]. This is consistent with the broader finding that personality accounts for a substantial amount of inter-individual variability in subjective wellbeing and happiness [18]. And when employees are senior leaders, such as executives, their personality shapes the culture of their organizations, impacting a large number of employees to determine their own career success [19]. Equally, leaders with problematic personality characteristics create high levels of turnover and underperformance in their teams and organizations [20].

It has been noted that the validity of personality traits as predictors of career potential—rarely above *r* = 0.30 for individual traits—tends to be underestimated by meta-analyses that fail to match personality traits to relevant career outcomes (e.g., extraversion and sales performance, conscientiousness and methodical task performance, openness and creative jobs, etc.) [21]. It is also likely that the effects of personality on career success are not linear, which suggests that previous findings have underestimated the strength of the relationship between the two variables [22]. For example, people who are too conscientious are more likely to display counterproductive levels of procrastination and obsessional work behaviors, as well as rigidly follow rules; while people with very low neuroticism levels may fail to experience the necessary levels of worry to perform well [23]. In addition, it is noteworthy that while individual traits may only be modestly associated with career outcomes, taking into account the overall contribution of multiple traits produces substantial multiple correlations. For example, the largest meta-analytic study on personality and leadership indicated that around 50% of the variability in leadership emergence and effectiveness can be attributed to the Big Five [24].

While the Big Five remain the universal currency in personality research, there is also compelling evidence for the validity of other, lower-order or higher-order, constructs to predict career-related outcomes [25]. For example, trait emotional intelligence, which is best understood as a meta-trait with each of the Big Five traits as one of its facets, is significantly correlated with job performance, job satisfaction, and leadership performance. Trait emotional intelligence does not, however, predict performance beyond the Big Five personality traits and other personality constructs [26]. Instead, recent meta-analytic evidence suggests that trait emotional intelligence is a meaningful construct to interpret the general factor of personality, which is indicative of social desirability or impression management [27].

Other personality traits that have been linked to career success include core self-evaluations [28], proactive personality [29], entrepreneurial personality [30], and integrity [31]. There is also compelling evidence for the importance of negative or counterproductive personality traits, such as the dark triad, as predictors of career derailment and failure [32]. Although some dark side traits have been found to contribute positively to individual career success [33], the dark triad traits Machiavellianism, Psychopathy and Narcissism also result in reduced quality of job performance as well as an increased risk for counter productive work behaviors [34].

An advantage of personality measures—over intelligence tests—is that they do not have an adverse impact [35]. This is particularly important when organizations are interested in increasing demographic diversity in their workforce [36]. Personality is not just an important predictor of career-related outcomes, it also enables higher levels of career success and job performance by increasing self-awareness and guiding developmental interventions, particularly in leaders [37]. Indeed, many organizations determine the specific training requirements of leaders based on their personality scores [38].

## 4. Real-World Misconceptions

If organizations were aware of the above-mentioned evidence, they would likely use personality and intelligence more often when making career-related decisions on their employees and leaders, including whom to hire, promote and develop. Note that employers often use indicators other than intelligence tests to infer individuals’ intelligence. For example, most employers take into account educational attainment, which correlates with intelligence at *r* = 0.50 [39]. Moreover, nearly all employers interview job candidates, and meta-analytic studies estimate that the correlation between job interviews and intelligence is around *r* = 0.40 [40]. In a similar vein, personality is often assessed via informal means or through methods other than psychometric tests. For example, the job interview is commonly used to infer candidates’ personality, and in turn their career potential [41].

However, there are some common misconceptions that determine the rather limited use of personality and intelligence measures in the workplace. First, organizations have a preference for competency models, despite their lack of scientific rigor compared to personality and intelligence. Originally developed to predict performance without the bias inherent in intelligence tests [42], competency approaches are strongly rooted in practice, often using less scientific methods and measurement approaches. Consequently, definitions of competency are incoherent, with persistent confusion around what competencies are and how they are measured [43]. Competencies needed for a job role are difficult to isolate, not least because they run the risk of over simplifying the behaviors required to succeed in a given role [44]. Rather than predictors of individual performance, competencies may be more useful as a way of describing and organizing internal resources, by establishing a link between job roles and employee characteristics [45].

Second, concerns about faking in self-reports, particularly high-stakes assessments, persist. Although there is evidence that faking has a negative impact on selection decisions such that, for example, those who fake show lower levels of performance later on [46], the problem is over estimated in practice. People do elevate their scores in application settings compared to low-stakes settings [47]. However, meta-analysis shows that personality tests retain their convergent and discriminant validity even in high stakes settings, and that social desirability does not predict overall job performance, or moderate the outcome-related validity of personality tests [48]

In addition, modern developments in scoring models, response formats and profiling methodologies are promising avenues for reducing the fakeability of personality tests themselves. Forced choice scoring models reduce the fakeability of self-reported tests in high stakes settings, whilst retaining the psychometric properties of conventional scoring models [49]. The same may be true for gamified formats, which could also integrate technology-based cheating detection systems commonly used in gaming [50]. However, being a relatively new trend in assessment, research on the fakeability of game-based assessments is outstanding.

Remote profiling of personality traits offers another possible avenue for reducing the fakeability of personality tests. Both social media profiles [51] and free text samples [52,53,54] accurately predict personality. Although arguably susceptible to social desirability, these data are harder to fake than self-reported questionnaires because they are collected over longer periods of time, and have lower face validity. In an organizational context, however, the use of social media data in particular is not without serious ethical concerns, calling into question the practicability of their use.

Third, intelligence tests, which largely avoid problems around faking by virtue of being ability tests, have lower face validity than other selection methods [55]. This affects applicants and employer acceptability of intelligence tests, leading organizations to shy away from their use despite their high predictive validity for job performance [56].

Fourth, an overemphasis on situational factors leads to the persistent underestimation of the role of dispositional effects. Decades of debate and research attest that both situational and personal aspects influence behavior [57]. In the work context in particular, a comprehensive analysis of existing research demonstrates that, for all of the Big Five traits, trait activation is of relatively higher importance in predicting job performance than situation strength, with over 50% stronger weightings in predicting performance [58]. This effect is even more prevalent in jobs with weak situational contexts, such as unstructured work and roles requiring decision making, and when certain traits are activated, such as Extraversion in sales roles, or Openness to Experience in innovation roles.

## 5. Stronger Together

Although there have been fairly consistent correlations between intelligence and Openness to Experience, including its facets [59], there are otherwise only trivial links between personality and intelligence, except when personality is evaluated through performance tests [60,61]. Where links exist, they are typically with lower level personality traits such as typical intellectual engagement or need-for-cognition and curiosity, which describe thinking dispositions or how individuals typically manage their cognitive capacity [62]. These lower level traits are strongly related to Openness, albeit describing thinking styles explicitly rather than including a preference for new experiences [63]. The overlap between Openness and thinking dispositions is consistent with the notion that personality determines the investment or development of adult intellect [64].

This means that personality and intelligence can be expected to explain separate aspects of career-related outcomes, implying incremental validity over each other [65,66,67]. For example, personality and intelligence combine to predict work-related expertise [68], job performance [7], leadership effectiveness [69], entrepreneurial success [70], hirability ratings [71], and manager ratings of competency [72].

The incremental validity of personality may be particularly high when predicting contextual performance factors [73]. It has also been pointed out that intelligence, which is measured via objective performance tests, is more predictive of maximum performance measures, while personality, which concerns predispositions and preferences, is more predictive of typical behaviors [74].

Looking at the combination of personality and intelligence in predicting job performance, a popular conceptual framework in Industrial and Organizational Psychology has been that personality and intelligence interact as predictors of job performance [75,76]. Within this framework, rather than personality and intelligence combining in an additive function to predict success, the function is thought of as multiplicative. Where there is an incremental increase in intelligence, an increase in motivation will be higher than when intelligence is lower. However, empirical studies exist that do not confirm this interaction, instead finding an additive effect of personality and intelligence in predicting work performance [77].

While personality and intelligence are not the only factors to predict and explain career-related outcomes, their combined explanatory power is hard to match—although vocational and career interests are almost as predictive [78]. For example, educational background and other human capital variables, which are more frequently taken into account for career-related decisions, such as hiring, explain significantly less variance in those outcomes [79].

## 6. The Future of Personality and Intelligence

In recent years, there have been many important technological advances to evaluate people’s career potential [80]. It is plausible to expect these developments to augment organizations’ focus on personality and intelligence, even if it means using non-traditional indicators for these constructs. For example, large organizations are awash with internal performance data to infer employees’ career potential, and personality and intelligence can provide theoretical models to explain those associations [81].

Although still in its early stages, there is growing interest in producing psychological insights from people’s digital data, such as their social media footprint [82]. Indeed, several independent studies have shown that machine-learning algorithms can be trained to translate Facebook and Twitter activity into relatively valid indicators of people’s personality and intelligence [83].

Similarly, there is increasing evidence that intelligence is accurately measured via games, or game-based assessments [84,85,86,87]. By applying machine learning algorithms to large numbers of data points collected during game play, such assessments achieve accuracy with shorter assessment time, making them particularly suitable to deliver comprehensive assessments of several characteristics such as personality and intelligence. Game-based assessments offer a better user experience, with higher satisfaction ratings and a heightened sense of flow from test takers [88,89]. In addition, game-based assessments improve engagement and motivation in test takers, and reduce levels of anxiety, resulting in better quality data across high and low stakes settings [90,91,92].

The notable advantage of game-based assessments is that they utilize machine learning algorithms, whilst avoiding the ethical implications of using social media footprints to retrospectively assess candidates. The data used for game-based assessments is generated by the candidate for the purpose of the assessment, with the candidates’ awareness of how it is being used. This is particularly relevant in applied settings where companies may want to avoid accessing social media data from candidates or employees. Although commercial game-based assessments of behavioral tendencies and preferences, or personality traits, exist, no evidence of their accuracy has been produced yet, with the exception of image-based assessments for creativity [93], as well evidence that personality can be inferred from video-gameplay [94].

Video and voice profiling offer another promising avenue for generating psychological insights without the need for lengthy assessments, or the ethical implications of using existing personal data generated for a purpose other than assessment. In addition, they show promising predictive validity in the organizational context: Audio and visual behavioral cues from interviewees and interviewers explain 36% of variance in hiring decisions [95].

Automated tools for predicting individual characteristics based on behavioral cues stem from the observation that human raters are able to make such inferences based on short video clips. For example, humans rate the personality and intelligence of individuals with moderate accuracy (*r* = 0.41 to 0.51 between stranger rating and the individuals’ intelligence test score [96]. Observable attributes such as hair color, stature and physical mannerisms indicate extraversion, whilst intelligence is inferred based on both physical and acoustic cues [97].

Automatic systems achieve similar, or higher, levels of accuracy by inferring personality based on social sensing, which is the use of machine perception and learning to analyze interpersonal behavior [98]. For example, non-verbal cues automatically extracted from online video resumes explain 27% of variance in first impression ratings of Extraversion, and 20% of variance in social and communication skills [99]. When taking into account both facial expression and vocal analysis, personality traits were classified correctly in 40–63% of cases, depending on the trait [100]. Non-verbal behavioral cues work best for predicting Extraversion [101,102], whilst the remaining Big Five traits are better predicted by verbal content [103].

Similarly, prediction models using speech clips achieve accuracies of 70–80% in classifying people’s Big Five personality traits [104,105], and speech signals such as rate, energy, pitch, silent intervals successfully distinguished between high and low extraverts in 86% of cases [106].

## 7. Conclusions

Both intelligence and personality consistently predict job performance, making them valuable metrics for organizations. Importantly, they also offer a theoretical framework and explanation for individual potential. New assessment formats offer promising avenues for promoting the use of intelligence and personality profiling in organizations by bringing the technological advances necessary to generate comprehensive profiles of intelligence and personality, without the burden of lengthy assessments. Modern formats may also help address some of the current barriers to use such as faking and acceptability to applicants. The role of Industrial and Organizational psychologists will be to uphold psychometric standards within new assessment formats, and to continually test the validity, reliability, and fairness of tools used to generate employee profiles, whether they be questionnaire- or algorithm-based.
